# Class II malocclusion associated with mandibular deficiency and maxillary and mandibular crowding: follow-up evaluation eight years after treatment completion

**DOI:** 10.1590/2177-6709.21.4.099-113.bbo

**Published:** 2016

**Authors:** Luís Antônio de Arruda Aidar

**Affiliations:** 1 Full Professor of Orthodontics, Universidade Santa Cecília (UNISANTA), Santos/SP, Brazil. Diplomate, Brazilian Board of Orthodontics and Facial Orthopedics.

**Keywords:** Angle Class II malocclusion, Corrective Orthodontics, Orthodontic anchorage, Stability.

## Abstract

This report describes the correction of a clinical case of malocclusion with anteroposterior discrepancy and transverse, sagittal and vertical deficiencies. A nonextraction technique was used to preserve space in the dental arches and control facial growth for the correction of the sagittal skeletal relationship and of overbite. The mechanics adopted efficiently corrected malocclusion: all functional and esthetic goals were achieved, and results remained stable eight years after treatment completion. This case was presented to the Committee of the Brazilian Board of Orthodontics and Facial Orthopedics (BBO) as part of the requirements necessary to obtain the BBO Diploma.

## RESUMO

O caso clínico apresentado descreve a correção de uma má oclusão com discrepância negativa e deficiências nos sentidos transversal, sagital e vertical. A mecânica de Cetlin foi utilizada visando à obtenção de espaços nas arcadas dentárias e o controle do crescimento facial, otimizando, assim, a correção sagital das bases ósseas e adequando o trespasse vertical. Essa mecânica foi eficiente na correção da má oclusão, atingindo todas as metas funcionais e estéticas, que continuaram estáveis oito anos após a conclusão do tratamento. Esse caso foi apresentado à Diretoria do Board Brasileiro de Ortodontia e Ortopedia Facial (BBO), como parte dos requisitos para a obtenção do título de Diplomado pelo BBO.

## INTRODUCTION

This report describes the case of a 13-year and 9-month-old boy, at the beginning of pubertal growth spurt, who first came to the office with his parents. His main complaint was that his maxillary teeth were "crooked." The examination of his medical history revealed that his health was good and that he had allergic rhinitis, under treatment with medication. His dental hygiene was satisfactory, and he did not report history of previous orthodontic treatment.

## DIAGNOSIS

**Figure 1:**
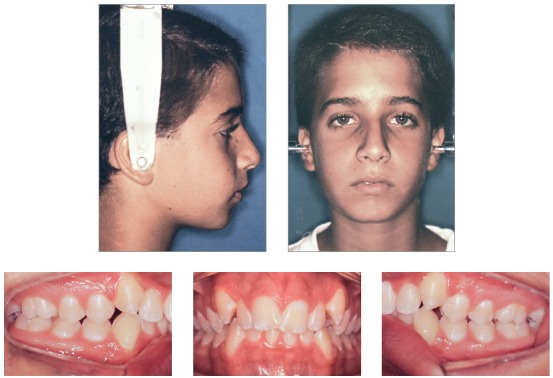
Baseline facial and intraoral photographs.

Figure 1 and Table 1 show the results of facial analysis. He had a convex profile (upper lip-S line = 1 mm; lower lip-S line = 2 mm) and no lip seal at rest. Nasolabial angle was normal, submental length was short, and the cervicomental angle was sharp. The lower third of the face was slightly larger than expected.

**Table 1:** Initial (A), final (B) and follow-up cephalometric values eight years after treatment completion (C).

	Measurements		Normal	A	B	C	Dif. A/B
Skeletal pattern	SNA	(Steiner)	82°	83°	81.5°	80°	1.5
	SNB	(Steiner)	80°	77°	78°	77°	1
	ANB	(Steiner)	2°	6°	3.5°	3°	2.5
	Wits	(Jacobson)	♀ 0 ±2 mm ♂ 1 ±2 mm	6 mm	4 mm	2.5 mm	2
	Angle of convexity	(Downs)	0°	13°	6°	2°	7
	Y-axis	(Downs)	59°	68°	67°	65°	1
	Facial angle	(Downs)	87°	80°	81°	82°	1
	SN-GoGn	(Steiner)	32°	30°	29°	27.5°	1
	FMA	(Tweed)	25°	27°	26°	23°	1
Dental pattern	IMPA	(Tweed)	90°	98°	102°	100°	4
	1.NA (degrees)	(Steiner)	22°	22°	29°	29°	7
	1-NA (mm)	(Steiner)	4 mm	4 mm	3.5 mm	4.5 mm	0.5
	1.NB (degrees)	(Steiner)	25°	24°	28°	24°	4
	1-NB (mm)	(Steiner)	4 mm	5.5 mm	6 mm	5.5 mm	0.5
	- Interincisal angle	(Downs)	130°	127°	118°	124°	9
	1-APo	(Ricketts)	1 mm	1 mm	3.5 mm	2.5 mm	2.5
Profile	Upper lip - S-line	(Steiner)	0 mm	1 mm	0 mm	-1.5 mm	1
	Lower lip - S-line	(Steiner)	0 mm	2 mm	1.5 mm	0 mm	0.5

**Table 2:** Initial (A), final (B) and follow-up measurements (C).

Measurement	A	B	C	Dif. A/B
Maxillary intercanine distance	34 mm	38 mm	37.5 mm	4
Mandibular intercanine distance	26.5 mm	28 mm	27 mm	1.5
Maxillary intermolar distance	51.5 mm	55 mm	57 mm	3.5
Mandibular intermolar distance	46 mm	48 mm	49 mm	2

**Figure 2:**
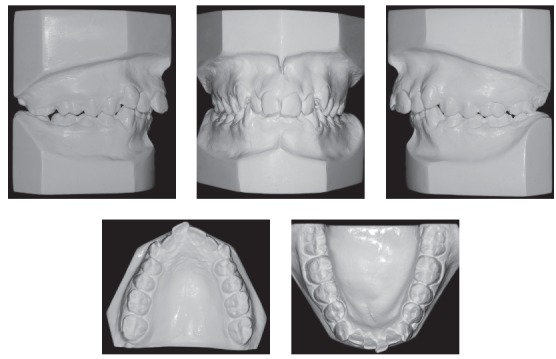
Initial casts.

Intraoral examination and study casts (Figs 1, 2) revealed that the patient had Angle Class II, Division 1 malocclusion, deep curve of Spee, mandibular incisor protrusion, 80% deep bite and 9-mm overjet. Maxillary and mandibular arches had transverse discrepancies, with slight maxillary and mandibular midline deviations. Tooth #12 was lingually inclined; teeth #13 and #23 had not erupted to normal level and were buccally inclined. Tooth size discrepancy was negative in both arches: -9 mm in the maxillary arch and -6 mm in the mandibular arch. Bolton's analysis revealed anterior mandibular tooth size discrepancy of 2.9 mm. 

**Figure 3:**
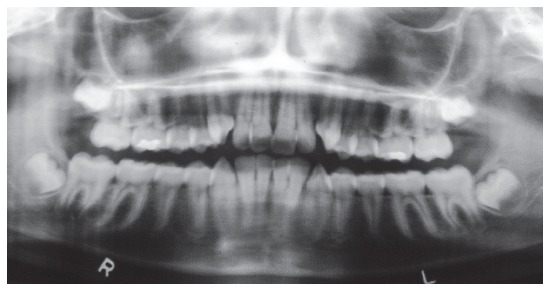
Baseline panoramic radiograph.

Panoramic radiograph (Fig 3) showed all third molars, with the mandibular ones proclined mesially. Other aspects, such as root contour, periodontal space and alveolar bone crest, were normal.

**Figure 4:**
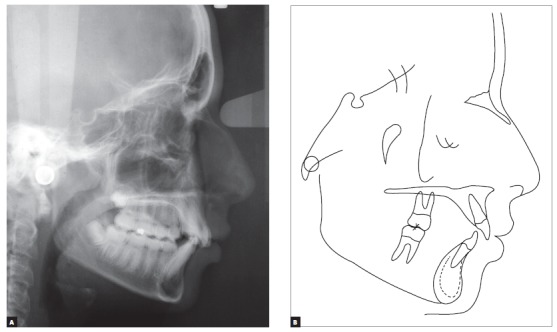
Baseline cephalometric profile radiograph (A) and cephalometric tracing (B).

Lateral radiograph and cephalometric tracing (Fig 4) revealed significant anteroposterior skeletal discrepancy (ANB = 6 degrees; Wits = 6 mm). The position of the maxilla relative to the cranial base was normal (SNA = 83 degrees), and the mandible was retruded (SNB = 77 degrees; facial angle = 80 degrees). The angle of convexity was large (13 degrees) due to the retropositioned mandible, and vertical growth pattern was balanced (SN-GoGn = 30 degrees; FMA = 27 degrees). Dental examination revealed that the position of maxillary incisors was good (1.NA = 22^o^ and 1-NA = 4 mm), and that the inclination of mandibular incisors was satisfactory, although they were slightly protruded (1.NB = 24^o^ and 1-NB = 5.5 mm). All cephalometric values are shown in Table 1.

Masticatory muscles and temporomandibular joints were painless at palpation and mandibular movement. 

## TREATMENT PLAN

A nonextraction protocol, as described by Cetlin,^s^ was chosen to treat the problems diagnosed. The purpose of this protocol was (1) to control growth of the posterior maxilla and, therefore, optimize anterior mandibular movement to achieve a harmonious profile and lip posture and a good skeletal relation during pubertal growth spurt; (2) to create spaces for correction of maxillary and mandibular discrepancies by expanding the dental arches in association with distal movement and rotation of maxillary molars and uprighting and rotation of mandibular molars; and (3) to correct deep bite by intrusion of mandibular incisors.

To correct maxillary crowding, distal movement and rotation of molars was planned, in association with dental arch expansion. First, a transpalatal bar would be placed on first molars to correct their rotation and create space in the maxillary arch. After that, a high-pull headgear would be used for anchorage at 250-g force on each side, to be worn for 10 hours a day, in association with a removable plate and another transpalatal bar placed on maxillary second molars. For the mandible, the use of a removable plate was planned, first on the mandibular first molar and, after fully erupted, on the maxillary second molars for uprighting and correction of rotation, as well as for lateral expansion of the mandibular arch.

After spaces were created and sagittal malocclusion corrected, a fixed orthodontic appliance (Roth prescription) would be placed in the maxillary and mandibular arches. After treatment completion and occlusion adjustment, a 0.032-stainless-steel wraparound removable retainer would be worn 20 hours a day for 12 months and overnight for other 12 months. A 0.028-stainless steel canine-to-canine retainer should be placed in the mandibular arch.

## TREATMENT PROGRESSION

For pedagogical purposes, impressions were taken at several treatment points to obtain dental casts and record the effects of treatment. After treatment completion, impressions of all dental casts were taken, and acrylic resin casts were fabricated. These acrylic casts were used for placement of appliances used in the nonextraction technique, in order to reproduce the exact clinical procedures at different treatment time points.

### Maxillary arch

As planned, spaces were obtained and preserved in the maxillary arch by means of a combination of a transpalatal bar on first molars to correct rotation, followed by a removable plate associated with extraoral anchorage on first molars and a transpalatal bar on second molars.^1^ ^,^ ^2^ In Class II malocclusions, in general, maxillary molars are mesially rotated. Therefore, transpalatal bares should be used initially to correct rotation, moving molars distally along the lingual root and, at the same time, expanding the dental arch with a slightly lingual crown torque. As first molars are rhomboid, their rotation opens spaces. Similarly, although second molars are usually triangular, a considerable amount of space will be created when their rotation is corrected.^2^

**Figure 5:**
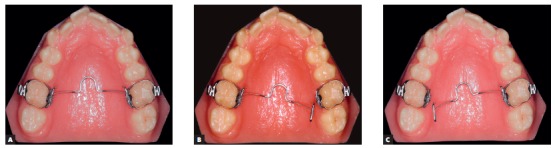
Transpalatal bar to correct maxillary first molar rotation (A); activation of arch at the two ends to promote rotation of first molars (B and C).

**Figure 6:**
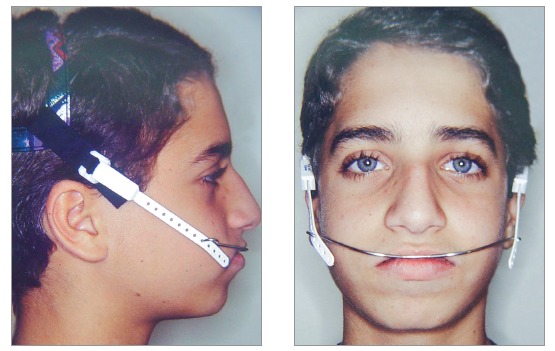
High-pull headgear.

**Figure 7:**
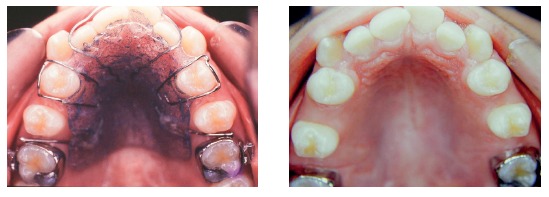
Removable plate with springs on maxillary first molars and spaces obtained in the maxillary arch.

Initially, a passive transpalatal bar was placed to connect the two first molars. Immediately after that, it was activated to rotate those teeth (Fig 5). After correction of molar rotation, a 1.5-mm expansion was incorporated. A high-pull headgear for overnight use was then placed for anchorage and force application of 250 g on each side (Fig 6). A removable plate with springs on first molars was also placed (Fig 7), and the patient received the recommendation to wear it continuously, only removing it for meals. The springs were activated at about 1 mm to 1.5 mm, measured on the occlusal surface of molars, and a distal force of about 30 g was applied. Vertically, the spring was placed at the most cervical position to reduce crown tipping, making sure that the gingival tissue was not affected. The combination of removable plate for crown tipping and extraoral anchorage with a short high-pull arm to elevate the line of action of force above the center of resistance of the tooth moved the molars.^1^ ^-^ ^4^

**Figure 8:**
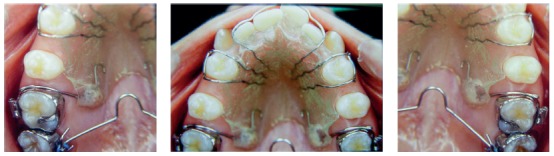
Removable plate on first molars associated with transpalatal bar on second molars.

**Figure 9:**
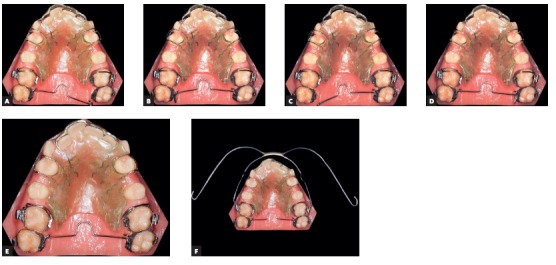
Activated transpalatal bar inserted on the right side of the cast to rotate and move left molar distally (A); inserted on the left side with no anteroposterior activation on the opposite side (B); activated and inserted on the left side of the cast to rotate and move left molar mesially and move right molar distally (C); inserted on the right side, no anteroposterior activation on the opposite side (D); removable plate and Adams clasp on teeth #14 and #24 and rectangular labial wire in the anterior region, not inserted to show 1-mm to 1.5-mm activation on first molars (A, B, C and D); removable plate and transpalatal bar inserted on tubes and headgear (E and F).

**Figure 10:**
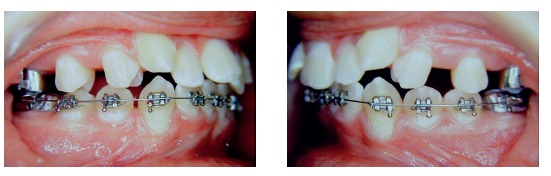
Overcorrected normal molar occlusion.

**Figure 11:**
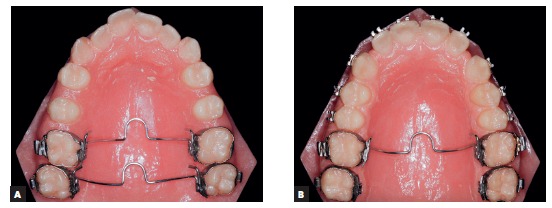
transpalatal bar on first and second molars after space creation and overcorrection of normal occlusion (A); maxillary leveling and alignment with transpalatal bar on first molars (B).

Another transpalatal bar placed on second molars was used in combination with the headgear and the removable plate to rotate and move second molars distally (Fig 8). The space created between first and second molars promoted distal movement of first molars, under the action of the removable plate and headgear. When activated on one side, the transpalatal bar rotated the tooth on that side and moved the opposite tooth distally. After distal movement of molar corrected Class II relationship, the transpalatal bar was activated on this side to correct rotation and move the opposite molar distally (Fig 9). The mesial force on the rotation side of the second molar was counter-balanced by forces applied by the headgear and removable plate. The transpalatal bar also controlled vertical growth of the maxilla. It was placed slightly away from the palate, with the Coffin loop turned mesially. The intrusive force of the tongue placed before the molar center of resistance promoted distal movement of molar roots.^2^ The maxillary fixed orthodontic appliance (Roth prescription) was placed only after molar occlusion was over corrected (Fig 10). In spite of that, the headgear and the transpalatal bar between fist molars were used all through alignment and leveling phases (Fig 11).

### Mandibular arch

As planned, the 1.2-mm stainless-steel removable plate, covered with a protective plastic tube on the anterior aspect, was placed. U-shaped folds worked as areas of adjustments and mesial stops for the molar tubes. The purpose of the removable plate was to expand the space in the mandibular arch and correct tooth position to enhance mandibular shape. The removable plate should be adapted to second molars whenever they can be included in treatment planning, so as to improve the vertical control of these teeth and correct potential rotations. Mandibular second molars should rotate until their lingual surfaces are parallel to each other, a position that has an important role in the shape of the arch.^2^

**Figure 12:**
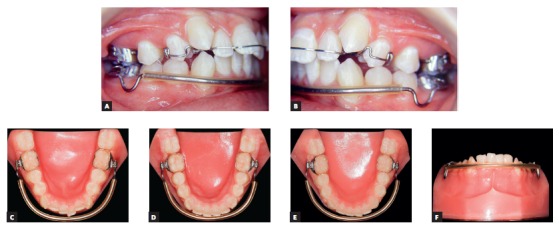
Removable plate (A and B); sequence of mandibular arch expansion, with rotation and uprighting of first molars and partial alignment of teeth after spaces were obtained (C, D and E); removable plate in the cervical region of mandibular anterior teeth to promote contact of lower lip with teeth and reduce buccal movement of incisors (F).

**Figure 13:**
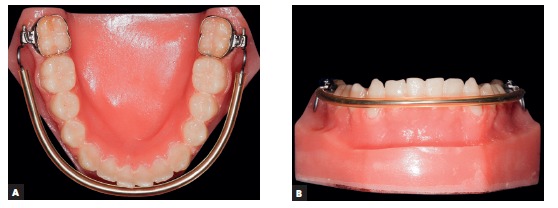
Removable plate on mandibular second molars for rotation and uprighting (A); in the anterior region, bumper is positioned in the cervical region of mandibular

Initially, as second molars were under eruption, the removable plate was placed between first molars to expand the mandibular arch (effect of the Fränkel appliance) and to promote the uprighting and rotation of mandibular molars following the movement of maxillary molars (Fig 12). Arch expansion is promoted by the tongue because of the removal of pressure applied by the buccinator on the buccal surface of mandibular teeth, as the appliance is adapted to promote a separation of 3 mm to 5 mm from the cheek.^2^ In the anterior region, the separation distance was planned to be only 2 mm, and the wire was placed at the height of the gingival margin, so that the lips kept contact with the buccal surface of mandibular incisors, which avoided imbalance between lip an tongue and kept incisors in right position.^1^ ^,^ ^2^ After the desired effect was achieved and second molars had fully erupted, the removable plate was transferred to second molars (Fig 13). 

**Figure 14:**
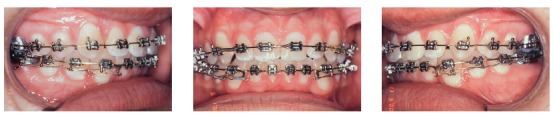
Fixed orthodontic appliance in the maxillary and mandibular arches, used to adjust occlusion.

**Figure 15:**
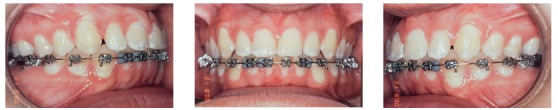
Bolton discrepancy, remaining spaces will be used to improve shape and increase mesiodistal diameter of teeth #12 and #22. incisors (B).

Another effect of this treatment was the control of vertical dentoalveolar growth of molars, which improved the forward movement of the mandible and correction of mandibular retrognathism.^2^ After spaces were created, a fixed orthodontic appliance (Roth prescription) was placed on all mandibular teeth for alignment, leveling and closing of remaining spaces. In this phase, overbite was corrected and the mandibular curve of Spee was reversed by means of a rectangular arch wire and resistant lingual torque in the anterior region (Fig 14). At the end of treatment, after occlusal adjustment, the patient was referred for restoration of the distal surfaces of maxillary lateral incisors to correct Bolton discrepancy detected during planning (Fig 15). During retention, a 0.032-stainless-steel wraparound removable retainer was placed in the maxillary arch, and the patient was instructed to wear it for 20 hours a day for 12 months and then overnight for other 12 months. A 0.028-stainless-steel canine-to-canine retainer was placed in the mandibular arch.

## RESULTS

**Figure 16:**
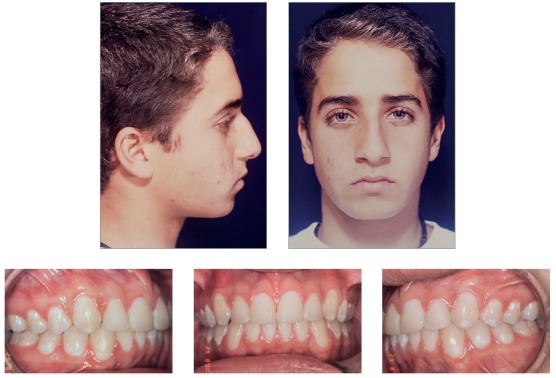
Final facial and intraoral photographs.

**Figure 17:**
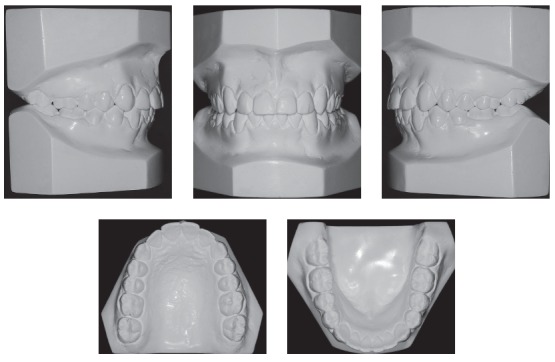
Final casts.

**Figure 18:**
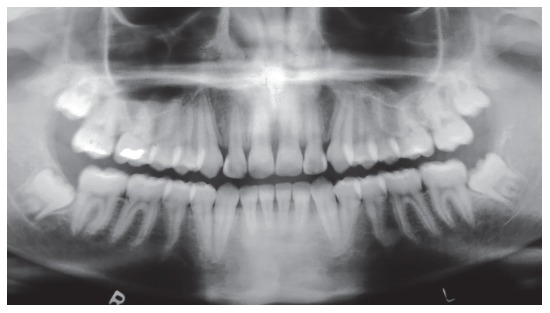
Final panoramic radiograph.

**Figure 19:**
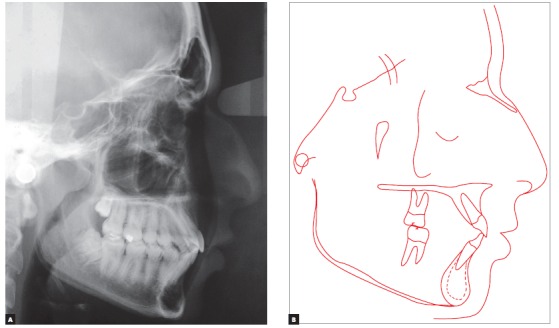
Final cephalometric profile radiograph (A) and cephalometric tracing (B).

At the end of treatment, analysis of patient's records (Figs 16 to 19) suggested that all initial objectives had been achieved. His facial profile became more harmonious after the lower lip moved forward, which defined a better position relative to the upper lip, and successful lip seal at rest.

Normal occlusion was achieved between molars and between canines, and overjet and overbite were corrected, resulting in excellent functional occlusion. Cephalograms revealed improvement of the anteroposterior relationship, with an 1.5-degree reduction of the SNA angle and an 1-degree reduction of the SNB angle, which resulted in a 2.5-degree variation of the ANB (from 6 degrees to 3.5 degrees) and a 2-mm-reduction of the Witts value (from 6 mm to 4 mm). There was also a 7-degree reduction of the convexity angle (from 13 degrees to 6 degrees). Vertically, there was excellent control of growth direction and a variation of only one degree in facial height parameters (SN-GoGn and FMA angles, and Y-axis). After treatment, the patient was referred to a specialist for extraction of third molars.

## POST-TREATMENT FOLLOW-UP

**Figure 20:**
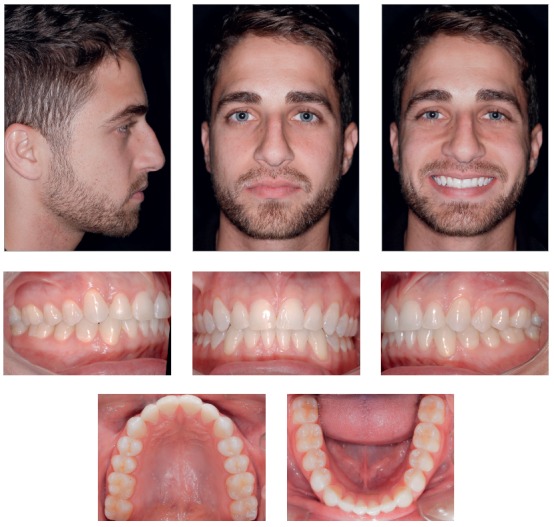
Facial and intraoral control photographs eight years after treatment completion.

**Figure 21:**
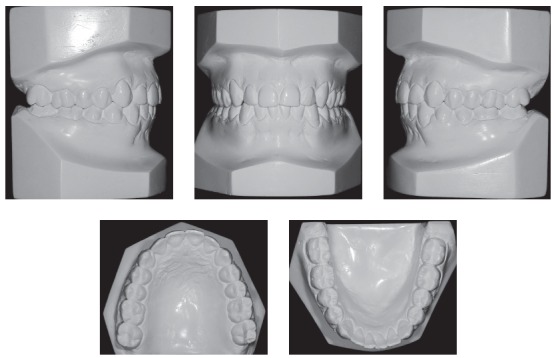
Follow-up casts eight years after treatment completion.

**Figure 22:**
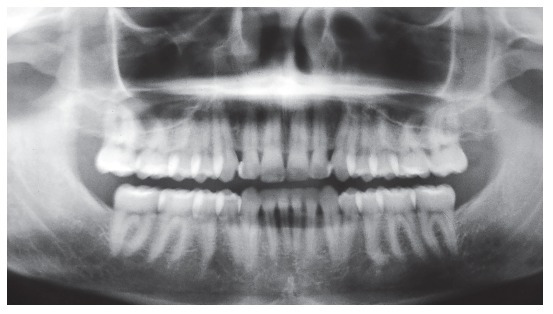
Follow-up panoramic radiograph eight years after treatment completion.

**Figure 23:**
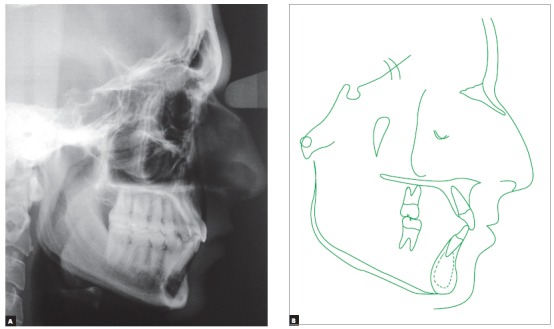
Follow-up cephalometric profile radiograph (A) and cephalometric tracing (B) eight years after treatment completion.

Patient's assessment eight years after the completion of active treatment revealed, according to the records obtained (Figs 20 to 23), that his facial profile was balanced, the facial thirds were proportional, and the smile was harmonious. Occlusion was stable, with discreet crowding in the mandibular anterior region. Functional occlusion was also balanced, with no problems in protrusive movements or right and left lateral movements.

The cephalogram described in Table 1 shows the comparison of values at baseline and at follow-up after eight years. There was significant improvement in the anteroposterior relationship and a 3-degree reduction of the ANB angle, a 3.5-mm reduction of the Witts value and an 11-degree reduction of the convexity angle. There was also vertical improvement, the mandible rotated counterclockwise, the SN-GoGN angle varied in 2.5 degrees and the FMA angle in 4 degrees.

**Figure 24:**
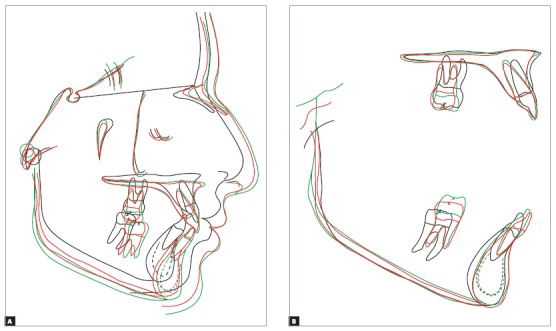
Total (A) and partial (B) comparisons of cephalometric tracings at baseline (black), treatment completion (red) and eight years after treatment completion (green).

Analysis of cephalometric superposition (Fig 24) revealed that the face moved forward and downward, a movement that was more evident when baseline and final findings were compared. At follow-up after eight years, the growth pattern was maintained, but at a lower rate. Individually, the sagittal movement of the maxilla was limited, whereas the mandible moved forward and downward between baseline and the end of treatment. During follow-up, the mandible continued growing, but at a lower rate. Facial profile improved, and lip position was more harmonious. Overjet and overbite in the incisor area were corrected. Partial superimposition showed that there was an increase in incisor proclination in the maxilla between baseline and the end of treatment, and this inclination remained unchanged at follow-up. The root moved buccally, and the crown remained stable. Molars moved distally, with a slight uprighting between baseline and the end of treatment. At follow-up eight years after treatment completion, there was a slight uprighting and mesial movement of molars. The evaluation of partial superimposition of the mandible revealed mandibular incisor proclination buccally, as well as slight uprighting during follow-up. The molar moved slightly mesially and, more significantly, vertically, following the vertical growth of the mandibular ramus at a greater rate when the baseline and the end of treatment were compared.

## FINAL CONSIDERATIONS

Advances in treatment mechanics have made it possible to obtain more space and to control facial growth better during correction of malocclusions with dental and skeletal discrepancies. In the case described herein, mandibular retrognathism was associated with maxillary and mandibular transverse discrepancies, in addition to great tooth size discrepancies in both the maxilla and mandible. The patient was at the beginning of pubertal growth spurt, had a good facial skeletal pattern and was highly motivated to cooperate when treatment to correct malocclusion was explained. The nonextraction technique was efficient to obtain spaces in the maxillary and mandibular arches, as well as to correct skeletal relationships. All functional and esthetic objectives were achieved, and the results were stable at follow-up eight years after treatment completion.
